# Biomarkers and Genetic Markers of Hepatocellular Carcinoma and Cholangiocarcinoma—What Do We Already Know

**DOI:** 10.3390/cancers14061493

**Published:** 2022-03-15

**Authors:** Jacek Baj, Łukasz Bryliński, Filip Woliński, Michał Granat, Katarzyna Kostelecka, Piotr Duda, Jolanta Flieger, Grzegorz Teresiński, Grzegorz Buszewicz, Marzena Furtak-Niczyporuk, Piero Portincasa

**Affiliations:** 1 Department of Anatomy, Medical University of Lublin, 20-090 Lublin, Poland; 55084@student.umlub.pl (M.G.); 58378@student.umlub.pl (K.K.); 56647@student.umlub.pl (P.D.); 2 Department of Forensic Medicine, Medical University of Lublin, 20-090 Lublin, Poland; 56749@student.umlub.pl (Ł.B.); 56724@student.umlub.pl (F.W.); grzegorzteresinski@umlub.pl (G.T.); g.buszewicz@umlub.pl (G.B.); 3 Department of Analytical Chemistry, Medical University of Lublin, 20-093 Lublin, Poland; j.flieger@umlub.pl; 4 Department of Public Health, Medical University of Lublin, 20-093 Lublin, Poland; marzena.furtak-niczyporuk@umlub.pl; 5 Clinica Medica “A. Murri”, Department of Biomedical Sciences & Human Oncology, University of Bari Medical School, 70124 Bari, Italy; piero.portincasa@uniba.it

**Keywords:** biomarker, genetic marker, hepatocellular carcinoma, cholangiocarcinoma, screening, diagnosis, therapy, treatment

## Abstract

**Simple Summary:**

Hepatocellular carcinoma and cholangiocarcinoma continue to remain a serious threat. In this review, we describe the most common biomarkers and genetic markers currently used in the diagnosis of hepatocellular carcinoma and cholangiocarcinoma. It can be observed that biomarkers and genetic markers might be applied in various parts of diagnosis including screening tests in a high-risk group, non-invasive detection, control of therapy, treatment selection, and control of recurrence. Also, it can be seen that nowadays there is a need for more specific markers that would improve the detection in early or very early stages of both types of cancers and further research should be focused on it.

**Abstract:**

Hepatocellular carcinoma (HCC) is the most common primary liver cancer with an increasing worldwide mortality rate. Cholangiocarcinoma (CCA) is the second most common primary liver cancer. In both types of cancers, early detection is very important. Biomarkers are a relevant part of diagnosis, enabling non-invasive detection and control of cancer recurrence, as well as in the application of screening tests in high-risk groups. Furthermore, some of these biomarkers are useful in controlling therapy and treatment selection. Detection of some markers presents higher sensitivity and specificity in combination with other markers when compared with a single detection. Some gene aberrations are also prognostic markers in the two types of cancers. In the following review, we discuss the most common biomarkers and genetic markers currently being used in the diagnosis of hepatocellular carcinoma and cholangiocarcinoma.

## 1. Introduction

**Figure 1:**
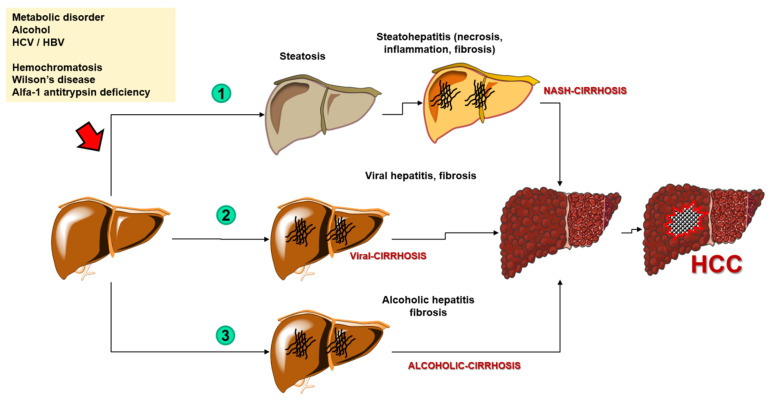
The process of carcinogenesis of the hepatocellular carcinoma. Abbreviations: HCC—hepatocellular carcinoma; NASH—nonalcoholic steatohepatitis; HCV—hepatitis C virus; HBV—hepatitis B virus.

Hepatocellular carcinoma (HCC) is the most common primary liver cancer (representing 75–85% of cases) and the fourth most common cause of cancer-related deaths in the world [1]. The mortality rate has been increasing for the past several dozens of years worldwide [2]. Although morbidity rate trends have recently been dropping in traditionally high-risk regions such as parts of Asia, they have lately increased in others such as North America and Europe [3]. The risk factors also depend on the region, but overall, the primary risk factor is liver cirrhosis (regardless of its etiology). More precisely, over a half (54%) of cases of HCC are caused by viral-related liver cirrhosis (including hepatitis B virus (HBV) with 33%, and hepatitis C virus (HCV) with 21%) while the rest of the cases include mostly alcoholic liver cirrhosis and non-alcoholic liver cirrhosis, the latter a consequence of the progressive form of non-alcoholic steatohepatitis (NASH) [1]. Other less common causes of HCC include various carcinogens such as nitrites, hydrocarbons, aflatoxins, and organochlorine pesticides, and genetic disorders including hemochromatosis, Wilson’s disease, and alfa-1 antitrypsin deficiency [4] (Figure 1).

Concerning diagnosis, abdominal ultrasound remains the first diagnostic and surveillance test for HCC. Although some cohort studies suggest computed tomography (CT) and magnetic resonance imaging (MRI) might have a higher early-stage detection sensitivity, there is still not enough evidence to evaluate whether these methods are clinically efficient and cost-effective. A major concern in the management of populations at risk of HCC is the early-stage sensitivity of diagnostic tests. In this context, the role of specific biomarkers is being actively investigated as alternative/complementary diagnostic tools for early diagnosis of HCC [1].

Cholangiocarcinoma (CCA) is the second most common primary liver cancer. The anatomical classification contains intrahepatic (iCCA) and extrahepatic (perihilar–pCCA and distal–dCCA) CCAcholangiocarcinoma. CCA shares several similarities with HCC by virtue of its increased incidence rate and its higher prevalence in Asian countries [2,5]. Mortality rates vary depending on the type of CCA, whether it comprises an increased amount of iCCA and decreased amount of extrahepatic CCA [6]. Overall, the prognosis of CCA is poor—a 5-year survival rate equals only 10%. Most of the cases of CCA are sporadic, although currently determined main risk factors include ‘biliary duct disorders, parasitic infections, and toxins and viral hepatitis B and C’, so that, along with liver cirrhosis, there is another resemblance to HCC. The main diagnostic and staging tests for CCA consist of high-quality cross-sectional imaging (CT or MRI), although diagnostic methods may be different for each type of malignancy [7]. These other methods include imaging tests such as abdominal ultrasound, positron emission tomography (PET), percutaneous transhepatic cholangiography, and endoscopic retrograde cholangiopancreatography (ERCP) or endoscopic ultrasound; or non-radiological methods represented by histological analysis and non-specific biomarkers. However, difficult early-stage detection and hard to define risk factors currently result in a high mortality rate. The diagnosis of CCA is often made late, limiting the effectiveness of surgical treatment that is the only efficient method of therapy [8]. Therefore, the need for specific and non-invasive biomarkers which may facilitate an early diagnosis and possible curative treatment of CCA has led to promising studies discovering such markers in various types of tissue and bodily fluids [9]. Due to the anatomy of the biliary tract system, and to the typically small size of CCA lesions, a procedure of obtaining tissue samples using ERCP presents several limitations. ERCP with intraductal ultrasonography (IDUS)-guided needle aspiration is linked to a risk of biliary tract perforation, and potentially severe biliary hemorrhage. The use of circulating nucleic acids, available in most biofluids is reported as a promising solution, providing tumor genomic information in a less invasive way, using plasma or bile samples [10].

In what follows, we discuss the current role of the most common biomarkers and genetic markers of HCC and CCA.

## 2. Search Strategy

We performed a literature review by Pubmed analysis based on international papers in the English language. In our analysis we used articles published between 1981 and 2021. The majority of articles used to describe clinical suitability were published over the past 10 years. We searched scientific works by using terms that included the keywords: biomarker; genetic marker; hepatocellular carcinoma; and cholangiocarcinoma, which were linked by “AND”. We focused on relevant international scientific journals available through Pubmed. In what follows, we discuss biomarkers and genetic markers of HCC and CCA, their application in screening tests, diagnosis, controlling therapy, and control of cancer recurrence. We report the current knowledge which originates from clinical trials and systematic reviews. Starting from a total number of 5381 articles about markers in HCC and 783 articles about markers in CCA we rejected all off-topic articles. We chose articles which contained information about the clinical suitability of markers. If papers contained the same kind of information, we chose the articles with the most current data. Finally, we collected 135 articles.

## 3. HCC Biomarkers

### 3.1. Alpha-Fetoprotein

Alpha-fetoprotein (AFP) is a glycoprotein entwined with the growth and development of HCC. It performs various functions in tumorigenesis—inducing malignant transformation as well as proliferation, migration, apoptosis, and immune escape [11]. Despite common use, it is characterized by poor sensitivity and specificity [12]. It can be used as a marker in screening tests for HCC for patients from at-risk groups—for example, HBV positive groups [13]. Also, a combination of AFP detection along with other markers seems to have a chance of increasing detectability of HCC–combination with alanine transaminase (ALT) and aspartate transaminase (AST) (that is liver function markers) [14,15], increasing detectability of AFP combined with Lens culinaris-agglutinin-reactive fraction of AFP (AFP-L3) and protein induced by vitamin K absence or antagonist-II (PIVKA-II) [16], as well as combined use of AFP, AFP-L3 and des-gamma-carboxy prothrombin (DCP) [17]. Detecting AFP in combination with the presence of the human cervical cancer proto-oncogene 1 (*HCCR-1*) appears to be beneficial in the detection of HCC less than 2 cm in diameter [12]. AFP is also being used in determining the probability of neoplasm recurrence in patients after a liver transplant [18], as well as a predictive value for postoperative prognosis after surgical liver resection—higher serum AFP concentration before the operation is a negative survival predictor after hepatectomy [19]. LI-RADS is a standardized diagnostic algorithm for liver imaging reporting. LI-RADS can also be used to decide on the type of treatment. According to the study by Centonze et al. tumors qualified as LI-RADS-5 are associated with higher median values of AFP than LI-RADS-3 and LI-RADS-4 nodules—higher LI-RADS class is associated with unfavorable pathological features. Furthermore, AFP is an independent risk factor for recurrence-free survival, log10AFP is an independent risk factor for cancer-specific survival, while LI-RADS did not have any significant impact on it [20].

### 3.2. Glypican-3

Glypican-3 (GPC-3) is a glycoprotein belonging to a family of proteoglycans containing heparan sulfate and expressed in 72–81% of HCC cases [21]. A high level of GPC-3 detected in blood serum is associated with poor prognosis and later stage tumor detection, vascular invasion, and metastases [22], also a rapid increase of GPC-3 expression is associated with the transition of precancerous lesions to HCC [23]. Detection of GPC-3 itself allows differentiation of HCC from healthy liver tissue, benign lesions, and liver cirrhosis [24]. Simultaneous detection of GPC-3 and AFP increases sensitivity and specificity of the test and consequently improves early diagnostics, as well as decreasing the risk of a wrong diagnosis [25]. In turn, PET imaging with the use of a probe specific for GPC-3 seems to be useful in HCC diagnostics [26]. Expression of GPC-3 by HCC cells also can also be used as a therapy, where induction of cytotoxic T lymphocytes, specific to GPC-3, by a vaccine reduces the risk of neoplasm recurrence and increases long-term survival [27,28]—for example, the use of antibodies against GPC-3 as cytotoxin carrying molecules, such as gemcitabine or sorafenib, which are supposed to target HCC cells [29,30], and natural killer (NK) cells specific for GPC-3 [31].

### 3.3. Osteopontin

Osteopontin (OPN) is a chemokine-like phosphorylated glycoprotein that remains inside of the cell or gets secreted. It has been observed to play a role in cell proliferation, inflammatory response, degradation of the extracellular matrix, angiogenesis, invasion, and metastasis [32,33]. It can be expressed in Kupffer cells, liver macrophages, and stellate cells but not in normal hepatocytes [34]. The increased expression of OPN has been correlated with digestive system neoplasms such as stomach, colon, and liver cancers, and pancreatic adenocarcinoma. Similarly, its relationship with tumor malignancy, invasion of surrounding tissue, and metastasizing ability [35] have been examined.

The combination of OPN detection and AFP has a greater diagnostic value than detecting these markers separately [36], and OPN also appears to be a useful marker in detecting AFP-negative HCCs [37]. A high level of OPN correlates with a poor prognosis—OPN plays an important role in tumor invasion and metastasizing, as it affects angiogenesis and inhibits the apoptosis of cancer cells [38]. Before performing the hepatectomy due to HCC, elevated OPN plasma levels suggest a poorer prognosis and shortened survival time after the surgery [39]. OPN also matters for treatment—its high levels suggest a cancer resistance to cisplatin [40].

### 3.4. Des-γ-Carboxy Prothrombin

Des-γ-Carboxy Prothrombin (DCP) is a prothrombin precursor expressed by HCC cells [41] that enables the differentiation of HCC from other chronic liver diseases such as hepatitis and cirrhosis [42]. It plays an important part in tumor development: it is being suggested that a positive DCP serum test indicates the occurrence of vascular invasion, intrahepatic metastases, and a high recurrence frequency [43]. Moreover, DCP serum concentration shows a positive correlation with tumor size and portal vein infiltration [44], with such infiltration being a negative predictor in HCC [45]. It is currently suggested that DCP plays a significant role in angiogenesis by stimulating the proliferation and migration of endothelial cells, which enables a vascular invasion of a tumor [45]. DCP is also useful as a marker in HCC diagnostics in combination with other markers such as AFP and AFP-L3 [46], in monitoring the treatment with transarterial chemotherapy [47], and also for choosing the therapy itself, since DCP antagonizes the apoptotic activity of gefitinib, thus leading either to a lack or to a poor response to the treatment [48]. According to a meta-analysis by Lai Q et al., DCP can be used in the qualification of a patient with HCC for liver transplantation. The high level of DCP before liver transplantation is associated with a higher risk of recurrence of HCC after transplantation [49].

### 3.5. AFP with a High Lectin Affinity

Alpha-fetoprotein with a high lectin affinity is the closest HCC-correlated fraction of AFP [4]. Its elevated levels before treatment indicate a poor prognosis, meaning it can be used as a predictor [50]. Used in combination with DCP and AFP it could be useful in diagnostics and screening tests for HCC [17]. Also, testing AFP-L3, AFP and golgi protein-73 (GP73) levels in venous blood collected from sublingual vein could be used as a screening test for HCC [51]. In turn, a combination of AFP-L3 and AFP or PIVKA-II has displayed high effectiveness in the detection of early HCC [52]. However, elevated levels of AFP-L3 occur not only in HCC, but also in severe hepatitis [53] yet, despite that, it appears to be a good marker for predicting reoccurrence, detecting small tumors, and detecting HCC in the early stage [54].

### 3.6. Golgi Protein-73

Golgi protein-73 (GP73) is a transmembrane glycoprotein located in type II Golgi apparatus [55], its increase of concentration occurs in such liver diseases as cirrhosis due to chronic HBV infection [56], focal nodular hyperplasia (FNH) [57], and HCC, in which elevated GP73 levels have been correlated with a shortened survival time—along with progressing cancerogenesis there occurs an increase in GP73 serum concentration [58]. The expression of GP73 and its levels also seem interesting when considering therapy for HCC. Rapamycin, an inhibitor of the mammalian target of rapamycin complex 1 (mTORC1), up-regulating GP73, appears to be a medicine that, by decreasing the expression level of GP73, causes inhibition of cancer development [59]. Also, suppression of GP73 expression has a positive impact on HCC by reducing metastasis and neoplasm invasiveness [59]. GP73 levels can also be used to evaluate a treatments success [60], to estimate the risk of possible complications after surgical removal of the liver, and can thus be used in patient selection [61].

## 4. Genetic Markers for HCC

### 4.1. MicroRNAs

MicroRNAs is a type of non-coding RNA that presents an 18–25 nucleotide length. It has a major role in regulating posttranscriptional gene expression, and recently it has been shown that miRNAs can indicate diagnosis and prognosis of different cancer types [62,63]. While obtaining miRNAs from tissue samples is an invasive procedure, it has been shown that, similarly to previous nucleic biomarkers, miRNAs can be available from serum [60].

It is now believed that incorrect miRNA expression plays an important role in the carcinogenesis of HCC [64]. Multiple types of miRNAs are potentially useful in HCC diagnostics, e.g., *miRNA-224*, whose expression level is higher in HCC cells than in normal hepatocytes and which affects the cell proliferation rate and cancer invasiveness. *Mir-224* concentration is higher in the serum of HCC patients and its levels correlate with survival time—the higher the concentration in the serum, the shorter survival time [65]. Overexpression of *microRNA-766* promotes proliferation and metastases, and also cell migration. The elevated expression level of *microRNA-766* is a negative predictor of HCC treatment, and *miRNA-766* itself could be potentially useful in treatment and diagnostics [66]. *MicroRNA-23* is an important oncogene for HCC progression and its high levels are detectable in patients’ serum so that it can be used as a marker of HCC. Produced in adipocytes, *microRNA-23* promotes cancer cells’ proliferation, and inhibition of its uptake might be a target for inhibiting the progression of HCC [67]. *MiR-494* promotes metastasizing and cancer invasiveness and it can also act as a biomarker for predicting the risk of reoccurrence in AFP-negative HCC patients. In this case, inhibition of expression also seems to be a target in the therapy of HCC [68]. In turn, measuring the levels of three miRNAs at once: *miR-10b*, *miR-106b*, and *miR-181a* is characterized by a high sensitivity and specificity, and this enables their usage for the HCC screening test [69].

### 4.2. Genetic Markers

Genomic analyses may enable better characteristics of a tumor, which will allow for treatment optimization of patients with HCC, and therefore research on HCC genetic markers is being conducted all over the world. The genes which are most subject to mutations in HCC are catenin beta-1 (*CTNNB1*) and tumor protein P53 (*TP53*) [70].

*CTNNB1* gene encodes β-catenin which plays an important part in cell adhesion and communication. A correlation between *CTNNB1* gene mutation and alcoholic HCC has been shown [70]. It has been stated that the inactivation of chromatin remodelers was dominant in tumors related to alcohol. An association of mutations in ribosomal protein S6 kinase A3-Axin 1 *(RPS6KA3-AXIN1*) and NFE2 like BZIP transcription factor 2-catenin beta-1 *(NFE2L2-CTNNB1*) genes suggest that Wnt/β-catenin signaling pathway could take part in hepatic cancerogenesis. It could affect oxidative stress metabolism and Ras/mitogen-activated protein kinase (MAPK) pathways [71]. *TP53* is a tumor suppressor gene encoding a protein that takes part in many cellular processes, especially in the activation of DNA repair mechanisms or in the induction of apoptosis in response to DNA damage. Whereas in the case of *TP53* mutation the cells with damaged DNA might avoid apoptosis and transform into cancer cells. A correlation has been shown between *TP53* mutation and HBV-related HCC. Inactivation of p53, either through mutation or binding to other viral and cellular oncoproteins, is often observed in neoplasms. Such interaction has been described in the case of p53 and the hepatitis B virus X gene (*HBx*) since *HBx* can inactivate the apoptosis which is mediated by p53 [72]. In tumors related to hepatitis B virus (HBV), there have also been shown suppressor properties of interferon regulatory factor 2 (*IRF2*), inactivation of which has led to a disabled function of *TP53* [71]. It has also been proven that *TP53* mutation can cause an inhibition of immune response in HCC [73]. The next genes regarding the frequency of mutations in HCC are *AXIN1* and AT-rich interaction domain 1A (*ARID1A*) [74].

*AXIN1* gene, being a negative regulator of the Wnt/β-catenin signal pathway, undergoes loss-of-function mutations. Research has shown that *AXIN1* mutations occur independently of Wnt/β-catenin pathway and they correlate with Notch and YAP pathways which can represent interesting targets in HCC treatment [75].

It has been found that decreased *ARID1A* expression level is associated with tumor progression, metastasis, and reduced survival [76]. It has been proven that patients with mutations in the *ARID1A* gene have a poor prognosis [77]. It also turned out that *ARID1A* mutation, along with *CTNNB1*, telomerase reverse transcriptase (*TERT*), and SWI/SNF related, matrix associated, actin dependent regulator of chromatin, subfamily A, member 2 (*SMARCA2)* mutations occur in alcohol-related HCC [78].

**Figure 2:**
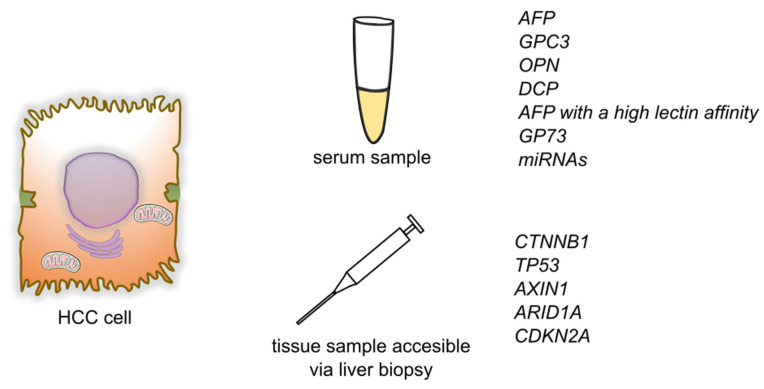
Summary of detection of biomarkers and genetic markers for hepatocellular carcinoma. Abbreviations: AFP—alphafetoprotein; GPC3—glypican-3; OPN—osteopontin; DCP—des-γ-carboxy prothrombin; GP73—Golgi protein-73; miRNAs—micro RNAs; *CTNNB1*—Catenin beta-1; *TP53*—Tumor protein P53; *AXIN1*—Axin 1; *ARID1A*—AT-rich interaction domain 1A; *CDKN2A*—cyclin dependent kinase inhibitor 2A.

Another gene undergoing a mutation in HCC is cyclin dependent kinase inhibitor 2A (*CDKN2A*). It encodes two proteins that work as cancer suppressors through cell-cycle regulation. The most common mechanism of inactivation of the *CDKN2A* gene in HCC is homozygous deletions [79]. Moreover, it has been stated that *CDKN2A* inactivation and fibroblast growth factor/cyclin D1 (*FGF/CCND1*) amplification correlated with poor prognosis [70] (Figure 2).

## 5. Biomarkers in CCA

### 5.1. Carbohydrate Antigen 19-9

Carbohydrate antigen 19-9/Cancer antigen 19-9 (CA19-9) is one of the two (besides carcinoembryonic antigen (CEA)) most widely used biomarkers of CCA [9]. It belongs to a larger group of mucinous markers, consisting of transmembrane protein skeleton and an extracellular part, which is built of highly glycosylated oligosaccharide chains. For a proper expression of the whole molecule, there is required a Lewis (*Le*) gene product–1,4-fucosyltransferase. Its presence is observed only in patients who have *Le* (a−b+) or *Le* (a+b−) blood groups. The entire absence of Lewis antigens occurs in approximately 6% of Caucasian and 22% non-Caucasian patients, which is associated with the lack of CA19-9 expression and false-negative results in this group [80,81,82]. CA19-9 constitutes a part of the secretion of biliary and pancreatic ducts cells, although it is also produced in the epithelia of salivary glands, stomach, colon, endometrium, and mammary glands [81]. Such an extensive CA19-9 expression indicates a wealth of potential causes of its increase in expression. These causes include: diseases of the bile ducts, pancreas, liver, spleen, salivary glands; endocrine disorders; lungs and airway, gynecological [81,83] and rheumatic diseases [84]; and many other neoplasms including liver, gall bladder, salivary glands, lungs, esophagus, stomach, colon, and female reproductive organs malignancies [80].

According to meta-analysis, CA19-9 sensitivity in the detection of CCA reaches 72% with a specificity of 84%. The sensitivity shows a certain variability regarding geography and it is slightly higher in Asian (74%) and American (71%) populations than European (62%) at a very similar specificity in all groups [85].

CA19-9 levels in serum may be used as a predictor in CCA. It has been observed that higher CA19-9 concentration (>103 U/mL) correlates with shorter survival (7.5 months versus 29.3 months), more frequent metastasizing, and the application of palliative therapy instead of surgical methods [86]. Furthermore, CA19-9 decrease of 20 and 50% relative to baseline, during a chemotherapy consisting of gemcitabine, is associated with longer survival [87,88] and increased preoperative concentration (>1000 U/mL) with shorter survival [89].

Like many other markers, CA19-9 presents a better diagnostic and differentiating (in this case for HCC) value when measured simultaneously with other markers such as CA125, CEA, and AFP [90,91].

### 5.2. Carcinoembryonic Antigen

CEA represents the second most frequently used biomarker for CCA. It is a largely glycosylated protein anchored in the cell membrane. The extracellular part is built of seven domains, each with the amino acid sequence similar to the immunoglobulin domain, and therefore CEA is a member of the immunoglobulin superfamily. Its production takes place mainly during fetal development and ends before birth, thus it is identified in a healthy person’s plasma at very low concentrations. It primarily contributes to proliferation, differentiation, cell adhesion, and suppression of cancer cells [92,93].

Increased plasma levels of CEA may be associated with various causes. They are observed in elderly patients, smokers, infectious diseases, gastric ulcer disease, inflammatory bowel disease, pancreatitis, hypothyroidism, hepatitis and liver cirrhosis, and benign breast tumors. Additionally, many malignant neoplasms are associated with elevated CEA levels, including malignancies of the colon, rectum, ovaries, uterus, lungs, esophagus, stomach, small intestine, liver, pancreas, breasts, spinal cord, and also osteosarcoma, retinoblastoma, multiple myeloma, lymphoma, malignancies of the urinary bladder and urinary tract, renal cell carcinoma and finally CCA. Given the multitude of causes of elevated CEA levels, it should not be used for the diagnosis on its own, but only in correlation with other clinical findings [94].

In the case of CCA, there is observed a higher CEA concentration. The sensitivity reaches 52%, and specificity 55% when measured for CEA alone. The use of CEA in combination with other markers such as CA19-9, matrix metalloproteinase 7 (MMP-7), and cytokeratin fragment antigen 21-1 (CYFRA 21-1) reaches a sensitivity level of 92% and specificity of 96%, making it a helpful diagnostic tool [95,96]

Beyond serum CEA measurements there is also the possibility for its detection in bile. It is there present in significantly higher concentrations and correlates with the type of bile duct disease (low CEA levels correlate with benign lesions, intermediate with primary sclerosing cholangitis, and high levels correlate with CCA and intrahepatic stones) [97].

CEA in combination with CA19-9 can be used as a predictor in patients with resectable and unresectable intrahepatic CCA. However, it has been shown that preoperative CEA concentrations do not correlate with overall survival [98].

### 5.3. CYFRA 21-1

CYFRA 21-1 is a fragment of cytokeratin 19 (CK-19)—a structural protein and fragment of intermediate filaments essential for maintaining epithelial cells stability. In healthy individuals, it appears in minimal concentrations as with other epithelial markers. As a result of enhanced protease activity in cancer cells, the degradation of CK-19 is augmented, which yields an increase of CYFRA 21-1 [99,100,101].

Elevated CYFRA 21-1 levels are observed primarily in lung and pleural neoplasms [102,103], squamous cell carcinomas of various areas of the body including lungs, esophagus, head, and neck [104,105,106] as well as stomach, colon, liver, breast, ovary, uterus, thyroid gland cancers, and pheochromocytoma. Moreover, elevated CYFRA 21-1 levels are present in infections, certain metabolic disorders, and chronic renal failure [107].

The overall CYFRA 21-1 sensitivity for biliary tract cancers is equal to 74.6% at a specificity of 84.6%. It should be noted that within this group, besides various types of CCA (iCCA, pCCA, dCCA), there is also included gallbladder cancer [101]. Certain research indicates lower values especially for the sensitivity (30%) [108]. The large meta-analysis points out that for iCCA type the sensitivity and the specificity amount to 81% and 86% respectively, whereby the marker itself is not appropriate for excluding this subtype of CCA [109].

Additionally, CYFRA 21-1 shows a predictive feature—its levels drop down after removing the cause of its growth, and they elevate at the disease reoccurrence. Furthermore, there is a correlation between its plasma levels and the stage of cancer and tumor aggressiveness. Patients with higher initial concentrations of CYFRA 21-1 present a shorter overall survival time [101].

At a concurrent usage of CEA and CA 19-9, the overall sensitivity and specificity reach 75.4% and 88.5% respectively. However, concurrent use of CYFRA 21-1, CEA, CA 19-9, and MMP-7 is more specific (96%) and sensitive [97]. As with other markers, CYFRA 21-1 should therefore also not be used alone [95,96,101].

### 5.4. Matrix Metalloproteinase 7

Matrix metalloproteinase 7 (MMP-7) or matrilysin belongs to a family of zinc-dependent proteinases produced by stromal cells which are capable of degrading the components of the extracellular matrix. It is the smallest of metalloproteinases and is expressed only in carcinoma cells. It plays a role in tissue invasion and metastasis [110].

Elevated MMP-7 levels are observed in the course of numerous neoplasms including breasts, ovaries, kidneys, colon, rectum, stomach, also squamous cell carcinoma of the esophagus, and pancreatic cancers [98,110].

The significant expression level of MMP-7 is also present in the case of biliary tract cancer. The research indicates a sensitivity ranging from 63 to 76%, and variable specificity oscillating between 46 and 78% depending on the cut-off value used [9,111,112].

It shows a feature of predictive value. Elevated MMP-7 levels correlate with a shorter survival time and poorer surgical treatment results [113]. Its increased expression correlates with a higher grade of malignancy and stage of the CCA [114].

### 5.5. Osteopontin

Apart from being a biomarker of HCC, OPN is also a biomarker of CCA. The sensitivity of OPN in detecting CCA equals up to 88% whereas the specificity is close to 100%, according to the meta-analysis [9].

The research results are inconsistent when it comes to the prognostic use of OPN. Its expression is related to tumor size, invasion, lymph node metastases, and grade of malignancy. It is also indicated that OPN could matter for an overall survival time [115,116,117,118] and that high OPN concentrations are a negative predictor for the patients who have undergone resection of intrahepatic CCA.

### 5.6. Interleukin 6

Interleukin 6 (IL-6) is an inflammatory cytokine with pleiotropic activity. From a variety of functions it stimulates B lymphocytes and increases production of acute phase proteins in the liver. High levels of IL-6 are also observed in case of many neoplasms [119]. CCA, as a tumor, in which the pathogenesis is highly related to an inflammatory process, is associated with elevated IL-6 levels. Moreover, it also shows a correlation with CCA size postoperatively [120,121]. Its sensitivity equals 73% at a specificity of 92% [122]. Furthermore, it is correlated with the level of differentiation of CCA [123]. It has been suggested that it should be used in combination with other markers, primarily with CA19-9.

### 5.7. New Potential Biomarkers of CCA

Among the new potential biomarkers, S100 calcium binding protein A6 (S100A6), Dickkopf WNT signaling pathway inhibitor 1 (DKK1), mucin 1 (KL-6), and spermatogenesis-associated protein 20 (SSP411) are mainly indicated. In case of these markers, new research is needed for establishing their clinical usefulness [9].

## 6. Genetic Markers of CCA

### 6.1. Circulating Nucleic Acids

Cell-free DNA (cfDNA) and Cell-free RNA (cfRNA) are potential diagnostic and prognostic markers that can be found in most biofluids. They appear in the circulating system in two ways—they can be actively exported or originate from dying cells [9]. cfDNA could potentially be a less invasive way to provide genetic information, compared to tissue sampling.

#### 6.1.1. Cell-Free DNA

cfDNA was first identified in blood samples in 1948 by Mandel and Métais [124]. It was used as a potential biomarker of neoplasm in the 1970s showing a link with neoplasm aggressiveness and size [9].

Symptoms of CCA and other biliary diseases are similar. Misdiagnosis of the mentioned diseases could be prevented by the usage of specific biomarkers. Wasenang et al. [125] investigated the potential serum of cfDNA biomarkers that could be used to differentiate CCA and other biliary conditions. In their study, they found that methylation levels of opioid binding protein/cell adhesion molecule like *(OPCML*) and homeobox D9 (*HOXD9*) differed significantly between CCA and other biliary diseases. Assessment of the biomarkers showed that sensitivity and specificity of *OPCML* were 80.0% and 90.0%, respectively, while in the case of *HOXD9* it was 67.5% and 90%, respectively. A combined marker between *OPCML* and *HOXD9* showed sensitivity and specificity of 62.50% and 100%, respectively (AUC: 0.812). The authors suggest that *OPCML* and *HOXD9* methylation measurement could be a beneficial method for minimally invasive, early differentiation of CCA and other biliary diseases.

In another study, a comparison was made between bile cfDNA and tissue sampling in detecting bile tract tumors mutations. Each of 10 patients (4 with gallbladder carcinoma (GBC) and 6 with CCA) provided a tumor tissue sample and paired bile sample. A panel of 150 tumor-related genes was used to analyze mutations individually in tissue and bile samples and then compared using targeted deep gene sequencing. In the aspect of single nucleotide variation (SVC) or insertion and deletion (Indel), the bile cfDNA showed high sensitivity and specificity (94.7%, and 99.9%, respectively) compared with the tissue sampling results. In the aspect of copy number variation (CNV) the cfDNA targeted deep sequencing had a sensitivity and specificity of 75.0%, and 98.9%, respectively. The authors suggest that targeted deep sequencing of bile cfDNA can be an accurate and sensitive method in detecting mutational variations of biliary tract carcinomas [10]. In a similar study, Driescher et al. [126] investigated the use of bile and serum cfDNA as a biomarker of extrahepatic CCA and pancreatic ductal adenocarcinoma (PDAC). Similarly, the results were compared to the results from matched tumor samples. In their study, sequencing of bile cfDNA showed a 96.2% concordance with tumor tissue samples with sensitivity and specificity of 100% and 100%, respectively. On the other hand, sequencing of plasma cfDNA revealed a concordance of 31.6%, compared to tumor tissue samples, and a concordance of 48%, compared to sequencing bile cfDNA. The authors suggest that sequencing of bile cfDNA might be a beneficial, ancillary diagnostic method in the diagnosis of pancreatobiliary cancers, while plasma cfDNA performs less reliably.

Wang et al. [127] evaluated the value of plasma CNV assays in the diagnosis of biliary tract carcinomas. Their prospective study was held among 47 patients with suspicious biliary lesions, of which 21 were diagnosed with CCA, and another 8 were diagnosed with GBC. In this study, CNV assays had a sensitivity and specificity of 89.7%, and 88.9%, respectively, while CA 19-9 had a sensitivity and specificity of 58.6%, and 72.2%, respectively. AUC of CNV assays was 0.91, which significantly outperformed CA 19-9, whose AUC was 0.62. Additionally, the authors showed that a higher CNV ratio was associated with decreased survival.

#### 6.1.2. Cell-Free RNA

Exosomes are a type of extracellular vesicles with a diameter of 30 to 150 nm containing molecules including DNA, mRNA, and different types of non-coding RNA, including piwi-interacting RNAs (piRNAs) [128]. Exosomes are excreted into the circulating system and thus are easily accessible during routine blood drawing [128].

piRNAs are a type of small, non-coding RNA with an ability to bind to PIWI family protein specifically. In their study, Gu et al. [128] investigated the potential role of plasma piRNAs in a diagnosis of the CCA and (GBC) in the group of 5 CCA and 4 GBC patients. It was shown that 694 and 323 piRNAs were upregulated in CCA and GBC, respectively, among which 163 molecules were upregulated concordantly. Thirty-six and 191 piRNAs were downregulated in CCA and GBC patients, respectively, among which 25 were downregulated both in CCA and GBC. On the other hand, *piR-4333713* was significantly downregulated for GBC and significantly upregulated in the CCA, suggesting differences between CCA and GBC in the exosomal signature. Additionally, the authors found that plasma levels of *piR-10506469* and *piR-20548188* decreased significantly after surgery for both CCA and GBC, and plasma levels of *piR-20548188* were correlated with CCA malignancy grade. Concluding, the authors suggest that exosomal piRNAs may have promising diagnostic values in the diagnosis of CCA and GBC.

#### 6.1.3. Cell-Free Long Non-Coding RNA

Recent studies have provided evidence that long non-coding RNAs (lncRNAs) play a role in carcinogenesis [129]. Bai et al. [129] in their study investigated the role of colon cancer-associated transcript 2 (*CCAT2*) in CCA. In their study, they found that *CCAT2* was overexpressed in 70.8% of CCA tissue samples, compared to adjected non-malignant tissue. The AUC for overall survival and progression-free survival was 0.702, and 0.715, respectively, suggesting that *CCAT2* may be useful as a prognostic biomarker. High expression of *CAAT2* was also linked to clinical features including microvascular invasion, differentiation grade, CCA progression, and metastasis. The authors suggest that *CCAT2* may be a promising prognostic factor and therapeutic target in the CCA.

In another study concerning lncRNAs as prognostic factors, Angenard et al. [130] showed that 9 of the concerned lncRNAs were significantly correlated with overall survival and disease-free survival. Four of them: (cyclin-dependent kinase 9 (*lnc-CDK9-1*), *XLOC l2 009441*, CDKN2B antisense RNA 1 (*CDKN2B-AS1*), HOXC13 antisense RNA (*HOXC13-AS*) were highly expressed in case of poor-prognosis iCCA, and the remaining 5 (long non-coding Coiled-Coil Alpha-Helical Rod Protein 1 (*lnc-CCHCR1-1*), *lnc-AF131215.3.1*, long non-coding Cbl Proto-Oncogene B *(lnc-CBLB-5*), COL18A1 antisense RNA 2 (*COL18A1-AS2*), long non-coding RELT like 2 *(lnc-RELL2-1*)) were upregulated in the case of better prognosis. The authors showed that *CDKN2B-AS1* was related to poor prognosis also in the other types of tumors, including HCC and kidney renal clear cell carcinoma.

#### 6.1.4. Micro RNA

Micro RNA has been studied as a marker of HCC, however, it has been also suggested as marker of CCA. *miR-21* is one of the best evaluated miRNAs in the CCA [9]. It has been shown that in the serum of CCA patients, the expression of *miR-21* was significantly upregulated and related to clinical features, including clinical stage, lymph vessel infiltration, and metastasis status. Compared with the low *miR-21* expression group, in the high *miR-21* expression group, values of overall survival and progression-free survival were significantly lower. Additionally, it has been found that *miR-21* levels decreased significantly after tumor operation. On the other hand, the receiver operating characteristic curve (ROC) analysis suggested that serum *miR-21* is defective in distinguishing CCA patients from healthy controls with an area under curve (AUC) value of 0.871. Its diagnostic results, including the sensitivity of 66.7% and specificity of 99.33%, were worse than CA 19-9 results (91.7%, and 99.33%, respectively). What is more, no significant difference was shown between CCA TNM Classification of Malignant Tumors (TNM) stage I patients and healthy volunteers or hepatolithiasis patients. The authors suggest that serum *miR-21* is an effective prognostic, but not a diagnostic marker of CCA [62].

*MiR-885-5p* is another miRNA molecule that is decreased as part of many malignancies, including pancreatic cancer and HCC [63]. It has a role in inhibiting tumor progression by reducing the expression of several genes including hexokinase 2, astrocyte elevated gene-1 (*AEG1*), and *CTNNB1* [63]. Lixin et al. [63] investigated the role of *miR-885-5p* in the tissue samples of 33 iCCA patients. Comparing with tissue samples of healthy individuals, *miR-885-5p* expression was aberrantly decreased in the CCA samples. Downregulation of *miR-885*-*5p* was also associated with clinical features including vascular invasion and lymph node metastasis and with shorter disease-free and overall survival. In a nude mice model with injected iCCA cells, overexpression of *miR-885-5p* reduced the incidence of lung and liver metastasis, while *miR-885-5p* inhibition acted inversely. The authors suggest that *miR-885-5p* could be a beneficial prognostic marker and therapy target in iCCA management.

In another study, Yao et al. [131] showed that, based on sequencing results, 83 of the miRs were upregulated and 45 of the miRs were downregulated in the samples of 36 CCA patients compared with 9 control samples. In particular, *miR-3913-5p* was highly expressed in the malignant tissues and high *miR-3913-5p* expression was identified as an independent prognostic factor of lower overall survival. It has also been shown that ring finger protein 24 (*RNF24*) and sialic acid binding Ig like lectin (*SIGLEC*), presumably involved in promoting proliferation and metastasis might be the target genes of *miR-3913-5p.*

Another miRNA molecule, a *miR-130a-3p* was studied by Asukai et al. [132]. Results of the study suggest that *miR-130a-3p* may be associated with gemcitabine resistance among patients with CCA. The authors also showed that peroxisome proliferator activated receptor gamma (*PPARG*) gene is a target of *miR-130a-3p* and pioglitazone, which is a PPARγ activator, alleviated gemcitabine resistance and had a synergic effect with gemcitabine.

### 6.2. Genetic Markers

The advent of new genome analysis technologies played a major role in the process of understanding tumor pathogenesis and heterogeneity. In a large study conducted among 489 CCA patients from 10 countries, Jusakul et al. [133] analyzed genetic features of CCA, including the whole genome sequencing (WGS) (*n* = 71), and DNA methylation (*n* = 138) assessment. In the WGS analysis, a total of 1,309,932 mutations were detected across 71 tumor samples, including 4541 nonsilient single nucleotide variations (SNVs) and 1251 nonsilient indels. On average, each tumor had 82 nonsilient mutations, including 64 SNV and 18 indels. Fluke-positive CCAs were significantly more plentiful in somatic mutations comparing with Fluke-negative tumors (median of 4700 vs. 3143 per tumor, respectively). Fluke infection was also correlated with poorer survival. Based on the results of their analysis, the authors suggest dividing CAA cases into 4 molecular clusters:

Cluster 1 was characterized by mostly Fluke-positive CCAs with hypermethylation of promoter CpG islands, enrichment of *TP53*, *ARID1A*, and BRCA1/2 DNA repair associated (*BCRA1/2*) mutations with reduced expression of Tet methylcytosine dioxygenase 1 (*TET1)*, and enhanced expression of enhancer of zeste 2 polycomb repressive complex 2 subunit (*EZH2)* and Erb-B2 receptor tyrosine kinase 2 (*ERBB2*) amplification.

Cluster 2 was also enriched in *TP53* mutations and *ERBB2* amplification, *CTNNB1*, Wnt family member 5B *(WNT5B*), and AKT serine/threonine kinase 1 (*AKT1*). It consisted of both Fluke-positive and Fluke-negative CCAs and represented a low level of methylation.

Cluster 3 showed specific upregulation of immune system genes, including immune checkpoint genes (programmed death receptor 1 *(PD-1*), programmed cell death 1 ligand 2 (*PD-L2*), and B- and T-lymphocyte-associated protein *(BTLA)*) and pathways related to the costimulation of T lymphocytes. Similar to Cluster 2, it was also characterized by a low methylation level.

Cluster 4 was characterized by enrichment of BRCA1 associated protein 1 (*BAP1*) and isocitrate dehydrogenase (NADP(+)) 1/isocitrate dehydrogenase (NADP(+)) 2 *(IDH1/2*) mutations and fibroblast growth factor receptor (*FGFR*) aberrations with upregulated expression of *FGFR1*, *FGFR2*, *FGFR3* and *FGFR4*. Similar to Cluster 1 its level of methylation was high, but, inversely, the methylation phenotype included CpG shore hypermethylation instead of CpG island hypermethylation, suggesting distinct mutational pathways.

Additionally, Clusters 1 and 2 were mostly represented by extrahepatic tumors, while Clusters 3 and 4 were characterized by intrahepatic malignancies almost entirely. Clusters 3 and 4 were associated with significantly better overall survival.

In another study, Lowery et al. [134] investigated molecular profiling of intrahepatic and extrahepatic CCA. An analysis carried out among CCA samples of 195 patients showed that in intrahepatic CCA the most commonly seen aberrations were *: IDH1* (30%), *ARID1A* (23%), *BAP1* (20%), *TP53* (20%), and *FGFR2 gene* fusions (14%). In the case of extrahepatic CCA, the most commonly found aberrations were: KRAS proto-oncogene, GTPase (*KRAS*), SMAD family member 4 (*SMAD4*), and serine/threonine kinase 11 (*STK11*) alterations. In addition, *CDKN2A/B* and *ERBB2* gene alterations were correlated with reduced overall survival and time to progression on first-line chemotherapy. Forty-seven percent of the patients showed somatic alterations with potential therapeutic value and thus 16% of the patients were enrolled in clinical trials molecular therapies.

**Figure 3:**
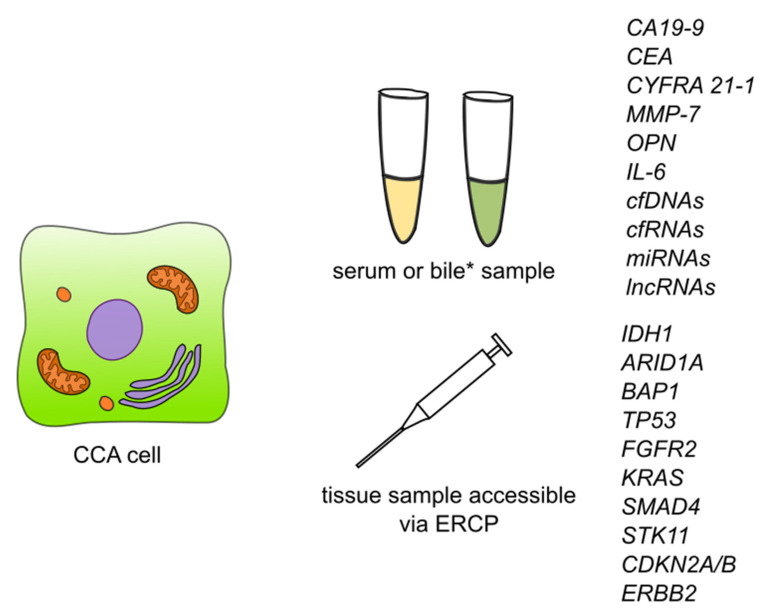
Summary of detection of biomarkers and some of the genetic markers for cholangiocarcinoma. Abbreviations: CA19-9—carbohydrate antigen 19-9/cancer antigen 19-9; CEA—carcinoembryonic antigen; CYFRA 21-1—cytokeratin fragment antigen 21-1; MMP-7—metalloproteinase 7; IL-6—interleukin 6; cfDNAs—circulating free DNAs; cfRNAs—circulating free RNAs; lncRNAs—long non-coding RNAs; ERCP—endoscopic retrograde cholangiopancreatography; *IDH1*—isocitrate dehydrogenase (NADP(+)) 1; *ARID1A*—AT-rich interaction domain 1A; *BAP1*—BRCA1 associated protein 1; *TP53*—tumor protein P53; *FGFR2*—fibroblast growth factor receptor 2; *KRAS*—KRAS proto-oncogene, GTPase; *SMAD4*—SMAD family member 4; *STK11*—serine/threonine kinase 11; *CDKN2A/B*—cyclin dependent kinase inhibitor 2A/cyclin dependent kinase inhibitor 2B; *ERBB2*—Erb-B2 receptor tyrosine kinase 2. * Detection of CCA biomarkers in bile has so far been described for CEA and cfDNAs.

Li et al. [135] carried out a study aimed at exploring the biological functions and prognostic biomarkers involved in CCA through transcriptional analysis. Thirty-three samples were obtained from CCA patients and 8 normal tissue samples. They discovered a total of 1463 differentially expressed genes, of which 267 were significantly upregulated and the remaining 1196 were significantly downregulated. According to Gene Ontology (GO) analysis, upregulated genes were enriched in ‘cadherin binding in cell-cell adhesion’, ‘extracellular matrix organization’ and ‘cell-cell adherens junctions’, while the downregulated ones were enriched in ‘oxidation-reduction process’, ‘extracellular exosomes’ and ‘blood microparticles’. Twenty-one of the genes were defied as hub genes including 8 upregulated genes and 13 downregulated genes. For all of the 21 hub genes, AUC was >0.900. Among upregulated hub genes, the expression level of *CDK1*, marker of proliferation Ki-67 (*MKI67*), DNA topoisomerase II alpha (*TOP2A*), and protein regulator of cytokinesis 1 *(PRC1*) were significantly negatively correlated to overall survival, while no similar correlation was found in remaining hub genes. Additionally, among downregulated hub genes, the expression of acyl-CoA oxidase 1 (*ACOX1*), apolipoprotein A2 (*APOA2*), apolipoprotein B (*APOB*), fibrinogen alpha chain (*FGA)*, and fibrinogen gamma chain *(FGG*) were negatively correlated with the tumor stage of CCA patients. The authors suggest that *CDK1*, *MKI67*, *TOP2A*, and *PRC1* could be used as prognostic biomarkers of CCA. The summary of detection of the biomarkers is showed in Figure 3.

## 7. Conclusions

In this non-systematic review, we presented basic information on biomarkers and genetic markers for HCC and CCA, which may be useful in early diagnosis, screening tests in high-risk groups, selection of appropriate treatment, and control of that treatment (Table 1 and Table 2). 

Despite rising knowledge in the domain of oncogenesis of HCC and CAA, which enable the discovery of and use in diagnosis, there is a need to assess novel biomarkers, which will be specific to these cancers and will ensure higher detectability and survivability of patients. It is also important that investigations proceed on the combination of two or more biomarkers, which could raise their sensitivity and specificity.

## Figures and Tables

**Table 1:** Some of the current and additional markers for early detection of HCC.

Marker	Pros	Cons	Notes	Reference(s)
AFP	Good for screening patients from risk groups	Low sensitivity	Established	[12,13,14,15,16,17,18,19]
GCP3	Negative prognostic value, Detection of GPC3 itself allows differentiation of HCC from healthy liver tissue, benign lesions, and liver cirrhosis, marker of malignant transformation	Specificity 72–81%	Established	[21,22,23,24,25,26,27,28]
OPN	Negative prognostic value, positive in AFP negative HCC		Experimental	[36,37,38,39,40]
DCP	Negative prognostic value, allows differentiation form other chronic liver diseases		Experimental	[42,43,44,45,46,47,48,49]
AFP-L3	Good for screening, detecting recurrence, negative prognostic value	Elevated in Hepatitis	Experimental	[17,50,51,52,54]
GP73	Negative prognostic value	Elevated in other diseases (HBV caused cirrhosis, focal nodular hyperplasia)	Experimental	[58,59,60,61]
miRNA	Negative prognostic value, possible screening value		Experimental	[64,65,66,67,68,69]

Abbreviations: AFP—Alpha-fetoprotein; GCP3—Glypicane-3; OPN—Osteopontin; DCP—Des-γ-Carboxy Prothrombin; AFP-L3—AFP with a high lectin affinity; Lens culinaris—agglutinin-reactive fraction of AFP, GP73-Golgi protein-73, miRNA-MicroRNA.

**Table 2:** Some of the current and additional markers for early detection of CCA.

Marker	Pros	Cons	Notes	Reference(s)
CA19-9	Negative prognostic value	Absent in Lewis(a-b-) patients, low specificity and sensitivity	Established	[80,81,82,85,86,87,88,89,90,91]
CEA	Negative prognostic value when used with other markers	Low specificity and sensitivity	Established.	[94,95,96,97,98]
CYFRA 21-1	Negative prognostic value	Low specificity and sensitivity	Established	[95,96,97,101,108,109]
MMP-7	Negative Predictive value, correlated with CAA stage	Low specificity and sensitivity	Experimental	[9,111,112,113,114]
OPN	High sensitivity		Experimental	[9,115,116,117,118]
IL-6	High specificity		Experimental	[120,121,122,123]
cfDNA	Possible diagnostic marker, correlated with tumor grade		Experimental	[10,125,126,127,131]
lncRNA	Shows prognostic value, correlated with tumor grade		Experimental	[129,130]
miRNA	Negative prognostic value	Bad diagnostic marker	Experimental	[9,62,63,131,132]

Abbreviations: CA19-9—carbohydrate antigen 19-9/Cancer antigen 19-9; CEA—Carcinoembryonic antygen; CYFRA 21-1—Cytokeratin fragment antigen 21-1; MMP-7—Metaloproteinase 7; OPN—Osteopontin; IL-6—interleukin 6; cfDNA—cel free DNA; miRNA—MicroRNA; lncRNA—cel free non-coding RNA.

## Abbreviations

ACOX-1	Acyl-CoA oxidase 1
AEG1	Astrocyte elevated gene-1
AFP	Alpha-fetoprotein
AFP-L3	Alpha-fetoprotein with a high lectin affinity, Lens culinaris-agglutinin-reactive fraction of AFP
AKT1	AKT Serine/threonine kinase 1
ALT	Alanine transaminase
APOA2	Apolipoprotein A2
APOB	Apolipoprotein B
ARID1A	AT-Rich interaction domain 1A
AST	Aspartate transaminase
AXIN1	Axin 1
BAP1	BRCA1 associated protein 1
BRCA1/2	BRCA1 DNA repair associated repair associated
BTLA	B- And T-Lymphocyte-associated protein
CA19-9	Carbohydrate antigen 19-9/Cancer antigen 19-9
CAA	Cholangiocarcinoma
CCAT2	colon cancer-associated transcript 2
CCND1	Cyclin D1
CDKN2A	Cyclin dependent kinase inhibitor 2A
CDKN2B	Cyclin dependent kinase inhibitor 2B
CDKN2B-AS1	CDKN2B antisense RNA 1
CEA	Carcinoembryonic antigen
cfDNA	Cell-free DNA
cfRNA	Cell free RNA
CK 19	Cytokeratin 19
CNV	copy number variation
COL18A1-AS2	COL18A1 antisense RNA 2
CT	computed tomography scan
CTNNB1	Catenin beta-1
CYFRA 21-1	Cytokeratin fragment antigen 21-1
dCCA	distal cholangiocarcinoma
DCP	Des-γ-carboxy prothrombin
ERBB2	Erb-B2 receptor tyrosine kinase 2
ERCP	endoscopic retrograde cholangiopancreatography
EZH2	Enhancer of zeste 2 polycomb repressive complex 2 subunit
FGA	Fibrinogen alpha chain
FGF	Fibroblast growth factor
FGFR1	Fibroblast growth factor receptor 1
FGFR2	Fibroblast growth factor receptor 2
FGFR3	Fibroblast growth factor receptor 3
FGFR4	Fibroblast growth factor receptor 4
FGG	Fibrinogen gamma chain
FNH	focal nodular hyperplasia
GO	Gene ontology
GP73	Golgi protein-73
GPC3	Glypican 3
HBV	Hepatitis B virus
HCC	Hepatocellular carcinoma
HCCR-1	human cervical cancer proto-oncogene 1
HCV	Hepatitis C virus
HOXC13-AS	HOXC13 antisense RNA
iCCA	intrahepatic cholangiocarcinoma
IDH 1/2	Isocitrate dehydrogenase (NADP(+)) 1/Isocitrate dehydrogenase (NADP(+)) 2
IDUS	Intraductal ultrasonography
IL-6	Interleukin 6
IRF2	Interferon regulatory factor 2
KRAS	KRAS proto-oncogene, GTPase
lnc-CBLB-5	long non-coding Cbl Proto-Oncogene B
lnc-CCHCR1-1	long non-coding coiled-coil alpha-helical rod protein 1
lnc-CDK9-1	long non-coding cyclin dependent kinase 9
lnc-RELL2-1	long non-coding RELT like 2
lncRNAs	long non-coding RNAs
MAPK	mitogen-activated protein kinase
miRNAs	MicroRNAs
MKI67	Marker of proliferation Ki-67
MMP-7	Matrix metallopeptidase 7
MRI	Magnetic resonance imaging
mTORC1	mammalian target of rapamycin complex 1
NASH	nonalcoholic steatohepatitis
NFE2L2	NFE2 like BZIP transcription factor 2
NK cells	Natural killer cells
OPN	Osteopontin
pCCA	perihilar cholangiocarcinoma
PD-1	programmed death receptor 1
PD-L2	Programmed cell death 1 ligand 2
PET	Positron emission tomography
piRNAs	piwi-interacting RNAs
PIVKA-II	protein induced by vitamin K absence or antagonist-II
PPARG	Peroxisome proliferator activated receptor gamma
PRC1	Protein regulator of cytokinesis 1
RNF24	Ring finger protein 24
RPS6KA3	Ribosomal protein S6 kinase A3
SIGLEC	Sialic acid binding Ig like lectin
SMAD4	SMAD family member 4
SMARCA2	SWI/SNF related, matrix associated, actin dependent regulator of chromatin, subfamily A, member 2
SNV	single nucleotide variation
STK11	Serine/threonine kinase 11
TERT	Telomerase reverse transcriptase
TET1	Tet methylcytosine dioxygenase 1
TOP2A	DNA topoisomerase II alpha
TP53	Tumor protein P53
WGS	Whole genome sequencing
WNT5B	Wnt family member 5B
